# SFGD: a comprehensive platform for mining functional information from soybean transcriptome data and its use in identifying acyl-lipid metabolism pathways

**DOI:** 10.1186/1471-2164-15-271

**Published:** 2014-04-08

**Authors:** Juan Yu, Zhenhai Zhang, Jiangang Wei, Yi Ling, Wenying Xu, Zhen Su

**Affiliations:** 1 State Key Laboratory of Plant Physiology and Biochemistry, College of Biological Sciences, China Agricultural University, Beijing 100193, China

## Abstract

**Background:**

Soybean (*Glycine max* L.) is one of the world’s most important leguminous crops producing high-quality protein and oil. Increasing the relative oil concentration in soybean seeds is many researchers’ goal, but a complete analysis platform of functional annotation for the genes involved in the soybean acyl-lipid pathway is still lacking. Following the success of soybean whole-genome sequencing, functional annotation has become a major challenge for the scientific community. Whole-genome transcriptome analysis is a powerful way to predict genes with biological functions. It is essential to build a comprehensive analysis platform for integrating soybean whole-genome sequencing data, the available transcriptome data and protein information. This platform could also be used to identify acyl-lipid metabolism pathways.

**Description:**

In this study, we describe our construction of the Soybean Functional Genomics Database (SFGD) using Generic Genome Browser (Gbrowse) as the core platform. We integrated microarray expression profiling with 255 samples from 14 groups’ experiments and mRNA-seq data with 30 samples from four groups’ experiments, including spatial and temporal transcriptome data for different soybean development stages and environmental stresses. The SFGD includes a gene co-expression regulatory network containing 23,267 genes and 1873 miRNA-target pairs, and a group of acyl-lipid pathways containing 221 enzymes and more than 1550 genes. The SFGD also provides some key analysis tools, i.e. BLAST search, expression pattern search and cis-element significance analysis, as well as gene ontology information search and single nucleotide polymorphism display.

**Conclusion:**

The SFGD is a comprehensive database integrating genome and transcriptome data, and also for soybean acyl-lipid metabolism pathways. It provides useful toolboxes for biologists to improve the accuracy and robustness of soybean functional genomics analysis, further improving understanding of gene regulatory networks for effective crop improvement. The SFGD is publically accessible at http://bioinformatics.cau.edu.cn/SFGD/, with all data available for downloading.

## Background

Soybean (*Glycine max* L.) is a major leguminous seed crop providing an important source of oil, and ranks first in oil production among the major oil seed crops [1]. In addition to its usage for human consumption, given its high content of essential fatty acids such as linoleic acid, soybean oil is an important renewable resource for chemical and biofuel production [2]. Increasing the relative oil concentration in soybean seeds is many researchers’ goal, using breeding methods and biotechnological strategies [3-5]. The key governing genes of seed oil biosynthesis in higher plants are those involved in the synthesis pathway of triacylglycerol (TAG), plastid fatty acids, endomembrane lipids and the storage process, which constitute more than 274 genes in soybean [6]. Several functional essays have shown that DGAT1 and DGAT2 have roles in seed oil accumulation [7-9]. Mutant alleles of FAD2-1A and FAD2-1B were combined to create soybeans with the high oleic acid trait [10]. Some important transcription factors (TF) regulating lipid metabolism and seed maturation have been reported, including basic leucine zipper (bZIP) and DNA binding with one finger (DOF) TF families [11,12]. However, most of these genes’ functions are not clear and the biosynthesis of soybean oil has yet to be elucidated.

With the availability of the soybean whole-genome sequence and large-scale application of high-throughput sequencing technology, research on soybean has made great progress. Numerous high-throughput data including genomics, transcriptomics, proteomics and metabolomics data are available for soybean. These data can provide valuable insights and improve soybeans if integrated and analyzed in a novel and comprehensive way. One of these ways is information mining from transcriptome data, given the overwhelming amount of such transcriptome data. The large amount of microarray expression data and deep sequencing transcriptomic data have allowed increasingly credible methods to be developed for generic networks, such as graphical Gaussian model networks through partial correlation [13], reverse engineering cellular networks using the ARACNE algorithm [14], networks based on improved PCC methods [15] and Bayesian networks [16]. In order to overcome the disadvantage of some functionally related genes with low PCC, a new variable ‘mutual rank’ or MR has been introduced [17,18], and co-expression networks for *Arabidopsis* and rice have been generated [19-21].

Some platforms and web services have been built, (e.g. SoyBase [22], SoyGD [23] and SGMD [24]), and other databases are shown in Table 1. These databases contain diverse information, such as genomic data, expressed sequence tags [24] and microarray expression data. Soybean transcription factors, transposable elements and partial ‘omics’ data are also integrated. Meanwhile, some useful tools have been developed, for instance, gene family browsing, BLAST searching and the gene pathway viewer–all providing good support for soybean research. However, the work on information mining and deep analysis for high-throughput transcriptome data including microarray and deep sequencing data is defective compared to the integration and simple analysis in previous studies in *Arabidopsis*[25,26]. As a useful tool, co-expression network was previously built [27], but it is also imperfect due to limitations on the types of microarray expression experiments used in its web services. In addition, although information concerning soybean metabolic pathways can be found in the Plant Metabolic Network (PMN) [28], a complete analysis platform of gene function annotation for the genes involved in the soybean acyl-lipid pathway is still lacking. Therefore, it is necessary to develop a powerful functional mining tool for soybean omics data and use it to predict candidate genes for molecular breeding related to oil.

**Table 1:** Summary of published soybean databases

**Database**	**Content**	**Source**
SGMD [24]	Genomic data, expressed sequence tags and microarray expression experiments, Proteomics of Oilseeds	http://psi081.ba.ars.usda.gov/SGMD/default.htm
SoyGD [23]	Soybean physical map and genetic map using Gbrowse as platform	http://soybeangenome.siu.edu/
Soybean Full-length cDNA Database [29]	40,000 full-length sequences of cDNA clones	http://rsoy.psc.riken.jp/
SoyDB [30]	Soybean transcription factors	http://casp.rnet.missouri.edu/soydb/
SoyTEDB [31]	Soybean transposable elements	http://www.soybase.org/soytedb/
SoyBase [22]	Comprehensive database for curated genetics, genomics, and related data resources developed by USDA-ARS	http://soybase.org/
LegumeIP [32]	Comparative genomics and transcriptomics database of model legumes	http://plantgrn.noble.org/LegumeIP/
PlaNet [27]	Whole-genome co-expression networks for seven important plant crop species	http://aranet.mpimp-golm.mpg.de/
SoyKB [26]	Integration of soybean omics data along with annotation of gene function and biological pathway	http://soykb.org/
SoyXpress [25]	Microarray expression data and expressed sequence tags [24]	http://soyxpress2.agrenv.mcgill.ca/
Phytozome [33]	Soybean genome sequence and gene annotation information	http://www.phytozome.net/soybean
Soybean eFP Browser [34]	Creates ‘electronic fluorescent pictographic’ representations of genes’ expression pattern	http://soykb.org/cgi-bin_new/efpWeb.cgi
SoyProDB [35]	Soybean seed proteins	http://bioinformatics.towson.edu/Soybean_Seed_Proteins_2D_Gel_DB/Home.aspx
GmGDB [36]	Soybean genome and gene models	http://www.plantgdb.org/GmGDB/
SoyPLEX [37]	Soybean gene expression resource	http://www.plexdb.org/plex.php?database = Soybean

Driven by this need, we developed a comprehensive database, the Soybean Functional Genomics Database (SFGD), which provides an integration and analysis platform for soybean ‘omics’ data and is a one-stop-shop resource for soybean acyl-lipid metabolism researchers. We hope it will improve the accuracy and robustness of soybean functional genomics analysis, and further speed up research on soybean yield and quality. This database contains 221 enzymes and more than 1550 genes involved in 15 soybean acyl-lipid metabolic pathways by combining data from the PMN [28] and prediction using *Arabidopsis* lipid-related genes. It also integrates genome and transcriptome data, e.g. coding genes, full-length cDNA and miRNA sequences, microarray expression experiments, deep sequencing experiments and single nucleotide polymorphisms (SNPs). In addition, a co-expression regulatory network including 23,267 soybean genes represented by 37,593 probe sets (the numbers do not match as one gene can be represented by one or more probe sets) with available microarray expression experiments (255 samples from 14 experiments) and 1873 miRNA–gene pairs in the network is included. There are some other function modules, e.g. a cis-element significance analysis toolbox for gene promoter sequences, an expression pattern search for microarray expression data so users can compare gene expression differences in various growth periods or under diverse stresses. There are also some general functions, such as browse, search and download of data. The platform is freely available at http://bioinformatics.cau.edu.cn/SFGD/.

## Construction and content

### Data sources

Soybean coding gene data and their annotations were downloaded from JGI (Joint Genome Institute) [6], which mainly included whole-genome sequences, coding gene sequences, protein sequences, gene location information and annotations. Full-length cDNA sequences were downloaded from the Soybean Full-Length cDNA Database [29]. We collected soybean miRNA and their precursor sequences from recently published literature and other repositories [38-45], and then mapped these miRNA precursor sequences against the soybean whole-genome sequence using the BLAST program (BLAST-2.2.19). Soybean probe set consensus sequences were downloaded from Affymetrix [46] and their annotations downloaded from B2G-FAR [47]. Microarray expression experiment data (CEL files) were downloaded from the Gene Expression Omnibus (GEO) [17] in the National Center for Biotechnology Information (NCBI) and normalized using Affymetrix GCOS software (TGT value: 500). We downloaded deep sequencing experimental data (fastq files) from NCBI Sequence Read Archive (SRA) with accession numbers SRP002082 [48,49], SRP002176 [50], SRP002459 [51] and SRP006767; SNP data (with 30-bp upstream and downstream flanking sequences) were collected from NCBI dbSNP, which included 17 wild and 14 cultivated soybean species [52]. Acyl-lipid pathway data were collected from PMN [28] and the ARABIDOPSIS ACYL-LIPID METABOLISM database [53]. The data source information is listed in Table 2. All the plant motifs were downloaded from the Plant Cis-acting Regulatory DNA Elements (PLACE) database [54], PlantCARE database [55], AthaMap database [56] and related publications [57]. There are total of 797 motifs and 66,207 soybean gene promoter sequences deposited in our database. Additional file 1: Table S3 lists 797 motifs and their occurrence frequency in these promoter sequences.

**Table 2:** Data source of SFGD

**Name**	**Number**	**Source**
Coding gene and annotation	66,207 genes, the items of cDNA, CDS and protein are all 75,778, respectively	ftp://ftp.jgi-psf.org/pub/JGI_data/Glycine_max/[6]
Full-length cDNA	37,870 (4708 full, 32,063 forward and 27,927 reverse sequences)	http://rsoy.psc.riken.jp/[29]
Consensus sequence	37,593	http://www.affymetrix.com/estore/index.jsp
Consensus sequence annotation	18,872 (including GO term, EC number and description)	http://bioinfo.cipf.es/b2gfar/home[47]
Microarray experiment	14 experiments, 245 samples	http://www.ncbi.nlm.nih.gov/geo
Deep sequencing data	Four experiments, 30 samples	http://www.ncbi.nlm.nih.gov/Traces/sra/sra.cgi?
MicroRNA data	229 (precursor and mature sequences)	http://bioinformatics.cau.edu.cn/PMRD/[58]
SNP	17 wild and 14 cultivated soybean species	[52]

### Microarray expression experiment normalization

Microarray expression experiment data (CEL files) were downloaded from GEO [17] in NCBI and normalized using Affymetrix GCOS software (TGT value: 500). Then we computed the average intensities ($\overset{¯}{x}$), standard deviations (stdev) and standard errors (stderr) using the following formulae:

$$\begin{array}{l} \bar{x}=\overset{n}{\underset{i=1}{\sum }}x_{i}\\ \text{stdev}=\sqrt{\frac{{\sum_{i=1}^{n}\left(x_{i}-\overset{¯}{x}\right)}^{2}}{n=1}}\\ \text{stderr}=\frac{\text{stdev}}{\sqrt{n}} \end{array}$$

Where x is intensity value and n is the number of replications.

### High-throughput sequencing data pre-processing

We downloaded deep sequencing experiment data from NCBI SRA, and removed the adapter sequence and low-quality bases at the 3’-end used the FASTX-Toolkit [59] if needed. We then checked read quality with FastQC - a quality control application for fastq files (http://www.bioinformatics.babraham.ac.uk/projects/index.html). Sequence reads that passed purity filtering were aligned with the whole soybean genome through bowtie [60] (version: 0.12.5) allowing for zero mismatches. The number of final reads of a DNA sequence was computed by the following formula:

$$n\;=\;{\text{log}}_{2}\;N\;+\;1$$

Where N is the number of raw reads mapped on the DNA fragment.

For each gene’s deep sequencing evidence of the 30 samples, each sample was normalized using the RPKM value [61].

$$\text{RPKM}=\frac{10^{9}\times C}{\mathrm{NL}}$$

Where C is the number of mappable reads falling into the gene, N is the total number of mappable reads in the experiment and L is the gene length in base-pairs.

### ZFE (Z-score for expression) and ZFM (Z-score for motif)

Z-score is a statistical measurement of the distance in standard deviations of a sample from the mean. Z-scores allow analysts to convert scores from different data sets into scores that can be accurately compared to each other [62], since they can act as a normalization method to eliminate the difference caused by background for a series of different experiments. For this reason, Z-score transformation statistics have been used in aspects of biology research, such as comparing gene expression between experimental and control groups in microarray analysis [63-66].

ZFE: we found that ZFE reflected the relationship of transcriptome change more accurately and visually than expression values (Additional file 2: Figure S2). Here we show the method used to generate the ZFE values from the average intensity values.

For each probe set in an experiment, if one of its average intensity values was greater than the Average Signal (A) (produced by GCOS) and max (average intensity)/min (average intensity) ≥ 2, and for datasets GSE7511, GSE7881, GSE8112, GSE9374, GSE10251 and GSE15100, we generated the ZFE using formula (1); for datasets GSE8432, GSE9730, GSE12300, GSE12314, GSE139631, GSE17883, GSE18822 and GSE20972, we calculated the ZFE using formula (2); otherwise ZFE = 0 [67].

(1) $$\text{ZFE}=\frac{\overset{¯}{x}‒\overset{¯}{\overset{¯}{x}}}{\text{stdev}}$$

(2) $$\text{ZFE}={\text{log}}_{2}\;\left(\frac{\overset{¯}{x_{T}}}{\overset{¯}{x_{c}}}\right)$$

Where $\overset{¯}{\overset{¯}{x}}$ is the average value of all $\overset{¯}{x}$ in an experiment, $\overset{¯}{x_{T}}$ is $\overset{¯}{x}$ of the treatment samples and $\overset{¯}{x_{c}}$ is $\overset{¯}{x}$ of the control samples.

ZFM: for one motif or set of motifs (in a gene or list of genes) submitted by users, the ZFM and P-value of each motif is calculated using the following method [68]. Initially, genome-wide genes are divided into several categories by promoter region length, e.g. genes with promoter region lengths < 500 bp, 500-1000 bp, 1-2 kb, 2-3 kb; secondly, ‘m’ submitted genes are classified with the same rules; thirdly, 1000 surrogate sets of ‘m’ promoters are obtained from groups with different promoter length, with sampling of the same proportions; and finally, for the motif_i_, the number of motif_i_ occurring in the promoters is N_motif_i_, the average occurrence frequency of motif_i_ in these 1000 sets is mean_motif_i_, and the standard deviation is stdev_motif_i_. The ZFM and P-value of motif_i_ are calculated only if N_motif_i_ > mean_motif_i_ using the following formula:

$$\text{ZFM}=\frac{N_{\text{motif}\_i}-{\text{mean}}_{\text{motif}\_i}}{{\text{stdev}}_{\text{motif}\_i}}$$

$$P‒\text{value}=1‒\text{pnorm}\;\left(N_{\text{motif}\_i},{\text{mean}}_{\text{motif}\_i},{\text{stdev}}_{\text{motif}\_i}\right)$$

Where pnorm() is the distribution function for the normal distribution in the R package, and the P-value cutoff is 0.1, which is decided with reference to the soybean genetic motif related experimental result, as well as the annotation of motifs in PLACE [54].

### MR

The MR [18] method was first used to construct a gene co-expression network in *Arabidopsis*[20]:

$$\mathrm{MR}=\sqrt{\mathrm{ab}}$$

For two genes (or probe sets) X and Y, first all microarray expression values are used to respectively calculate all genes’ PCC values for X and Y. Then PCCs are respectively sorted from large to small for X and Y; then ‘a’ (start from 1, with step size of 1) is used to mark the position of Y in X’s list, and similarly, ‘b’ marks the position of X in Y’s list. For the hub gene, the network shows the top 10 co-expressed genes according to the arrangement of MR from small to large in the first level; for the second level, the top five genes that co-expressed with first level genes are selected; and the selection of genes in the third level follows the same rules as for the second level. The threshold of MR is in reference to the already published *Arabidopsis* co-expression network [19].

### miRNA:target alignment criteria

In plants, miRNAs generally precisely direct their mRNA targets for endonucleolytic cleavage [69-71]. To predict potential targets of the miRNAs in this study, we developed a set of computational ‘criteria’ for soybean miRNA-target interaction based on previous research [69,72]. The ‘criteria’ as following: (a) The mismatch of miRNA:target must be ≤ 4 and the mismatch in the first half-pair must be ≤ 2.5 (G:U = 0.5); (b) The continuous mismatch must be ≤ 2 and continuous mismatch between 2 and 12 bp must be ≤ 1; (c) The bases 11 and 12 must be a perfect match; and (d) The minimum free energy (MFE) of miRNA:target must be < 75% compared with when the miRNA perfectly matched with its target. With these criteria, we successfully predicted 1873 miRNA-gene pairs.

## Utility and results

SFGD uses Generic Genome Browser (Gbrowse) as an integration platform for soybean transcriptome data (Table 2) and generates some data mining tools, such as ‘gene co-expression network’, ‘pathway’ and ‘motif’. The tool ‘gene co-expression network’ aims to display a gene’s co-expressed genes and miRNA:target interaction; ‘pathway’ is a functional tool containing soybean acyl-lipid metabolic data; ‘motif’ is a tool for searching and analyzing significant cis-elements in one or more genes; and ‘pattern’ is a tool for searching tissue/time specific genes based on microarray expression experiments (Additional file 3: Table S1), and some other function modules such as BLAST and general search.

### Gbrowse: a repository of the transcriptional information

Gbrowse is a genome viewer. Soybean protein coding genes, full-length cDNAs, miRNA precursors, probe set consensus sequences, microarray expression experiments and deep sequencing data have been integrated into Gbrowse with clickable links to a new webpage. This webpage shows descriptions of each keyword: e.g. ‘gene detail information web page’ shows the co-expression gene network, microarray expression profile, deep sequence evidence and coding sequence; for ‘microarray expression data’, a diagram displays the other probe sets related to it with PCC > 0.7 or PCC < -0.65.

The coding gene’s result page includes gene annotation, the list of co-expressing genes, 14 microarray expression experiments [only if there is a probe set(s) in its context] and deep sequence evidence of it in the 30 deep sequencing samples. Following are the gene’s genome location information and sequences (including promoter, cDNA and protein sequences).

For the microarray expression profile section (e.g. ‘GmaAffx.88235.1.S1_at’), we have integrated 14 experiments in total. Each has horizontal bars according to the experiment’s treatment and control expression level generated using ZFE (red bars represent up-regulated and green bars down-regulated, Additional file 4: Figure S1). For example, if users click horizontal bars below ‘GSE7511’ (‘Expression data from soybean seed compartments with embryos at the heart stage’), it will show gene Glyma15g34770’s (represented by probe set ‘GmaAffx.88235.1.S1_at’) expression profile in different tissues of soybean seed. With ‘GmaAffx.88235.1.S1_at’ is included a diagram generated using expression values and standard errors, values table display and the probe sets most positively and most negatively correlated (PCC > 0.7 and PCC < -0.65, respectively). Users can get all these probe sets’ expression values and line charts produced by expression values and ZFEs (Additional file 2: Figure S2) and send the probe set list to agriGO [73] for gene ontology (GO) enrichment analysis. Users can also get the promoter sequence of genes that are positively correlated with this gene and scan the cis-elements contained for significance analysis.

miRNAs are ~21-nt-long endogenetic non-coding small RNAs that function as post-transcriptional regulators in eukaryotes [74]. All miRNAs in SFGD came from recently published literature [38-45]. Here, soybean miRNA information is integrated into PMRD (Plant MicroRNA Database) format [58], which mainly includes four regions: precursor, mature, target and reference regions.

The repository also has 30 RNA-seq samples that cover various tissue types and stress conditions. This can be used for deep sequencing evidence of one gene’s expression in particular tissues or at particular time points.

For other content in Gbrowse–such as full-length cDNA, probe set consensus sequence and SNP information–users can obtain their description and related information by clicking the corresponding icons.

### Gene co-expression network

We generated a soybean gene co-expression regulatory network based on 255 samples from 14 microarray expression experiments using PCC and MR [18]. After converting the probe sets to the corresponding genes, we obtained a gene co-expression network including 23,267 genes represented by 37,593 probe sets (one gene may be represented by one or more probe sets).

There are 240,496 edges left after setting the MR threshold ≤ 30, giving 20.7 edges for each gene on average–Additional file 5: Table S2 lists the top 10 genes with the greatest number of edges as well as the genes’ annotations. Cytoscape Web software [75] is used to display the gene network, and for each gene (central node), all its co-expression genes are sorted according to MR values and several with the top values are displayed. There is a recursive search using this process for the second and third levels. For the first level [genes (subprime nodes) immediately linked with the central gene], the top 10 MR-value genes are displayed. For the second level (genes directly linked with first level genes, linked with central node through subprime nodes) the top 5 MR-value genes for each subprime node are shown. In the third level, although we collected all top 10 MR-value genes of second level genes, only those which have previously displayed in the first and second levels remain, because other genes are not likely to be closely related to the central gene. A table is also generated to show all genes and gene annotations appearing in the Cytoscape Web file–users can use these genes to scan their promoter motifs and analyze their significance using the textbox at the bottom of the web page.

The soybean miRNA: target information was also integrated into the network. Soybean miRNA:target pairs were predicted using in-house programs with all miRNAs and mRNA genes deposited in our database, then we compared our results with psRNATarget [76]. Finally we separated the results into two parts: high credibility miRNA:targets (338 pairs, predicted by both programs) and low credibility miRNA:targets (1535 pairs, predicted by one of the programs).

### Acyl-lipid metabolic pathways

Due to the importance of oil crops, the need for acyl-lipid metabolism research is self-evident. Through integrating data from PMN [28] and prediction using *Arabidopsis* lipid-related genes, we obtained 221 enzymes and more than 1550 genes involved in 15 soybean acyl-lipid metabolism pathways (Table 3). The pathway card page provides a user-friendly view of lipid metabolic pathways and information about metabolism alias names, mass-to-charge ratios and chemical formulae. Users can also search for their lipid of interest on the lipid page.

**Table 3:** Soybean acyl-lipid metabolism pathway

**Lipid metabolism pathway**	**Enzyme numbers**	**Gene numbers**
Fatty acid synthesis	20	88
Fatty acid elongation, desaturation and export from plastid	17	55
Triacylglycerol biosynthesis	14	126
Triacylglycerol and fatty acid degradation	18	148
Eukaryotic galactolipid and sulfolipid synthesis	17	63
Prokaryotic galactolipid, sulfolipid, phospholipid synthesis	25	112
Eukaryotic phospholipid metabolism	18	97
Mitochondrial phospholipid metabolism	9	62
Sphingolipid synthesis and transport	22	61
Mitochondrial lipoic acid synthesis	13	46
Wax synthesis and transport	22	207
Cutin synthesis and transport	7	63
Suberin synthesis and transport	17	279
Oxylipin metabolism	21	120
Choline synthesis	8	23

### Motif scan

If the user’s input is a fasta format sequence of a promoter, all motifs appearing in the sequence are listed, and these motifs’ occurrence frequencies counted using the in-house program.

### Cis-element significance analysis

For each gene’s promoter sequence or a sequence submitted by the user, all the potential motifs are scanned. If a list of gene names is submitted, all motifs appearing in their promoter region will be scanned. The platform also offers the result of significant motifs in promoters of these genes.

### Expression pattern

In order to find genes’ specific expressions under specific conditions, we selected four experiments with different tissues and stress conditions. GSE7511, which contains 23 samples to identify all genes active in 10 different compartments, was isolated using the Leica AS LMD system from heart-stage seed. GSE7881, includes 18 samples, identified all genes active in cotyledon-stage seed within nine isolated compartments. GSE8112, contains 34 samples obtained from early maturation stage of seed, which was isolated within 17 compartments. GSE8432 contains 27 samples obtained from a soybean plant PI200492 with two treatments: HW94-1 (*Phakopsora pachyrhizi* isolates, which produces a resistant reaction in the host) or TW72-1 (*P. pachyrhizi* isolates, which produces a susceptible reaction in the host) at four time points (6, 12, 24 and 48 h post inoculation). Furthermore, we developed two function tools in the corresponding web page: ‘Expression pattern search’ and ‘Tissue/time specific search’.

For the ‘Expression pattern search’ function, selections submitted by users are converted to corresponding values – high (or up-regulated), even and low (or down-regulated) represented by 1, 0 and -1, respectively–then PCC is calculated between users’ selections and ZFE of data in our database, using a PCC threshold of 0.7.

The ‘Tissue/time specific search’ toolbox only returns probe sets that are highly expressed in the specific condition according to the ZFE set up by users. The resulting web pages for both ‘Expression pattern search’ and ‘Tissue/time specific search’ also include links to ‘agriGO’ [73] for GO enrichment analysis and ‘motif scan’ function for cis-element significance analysis as described above.

**Figure 1:**
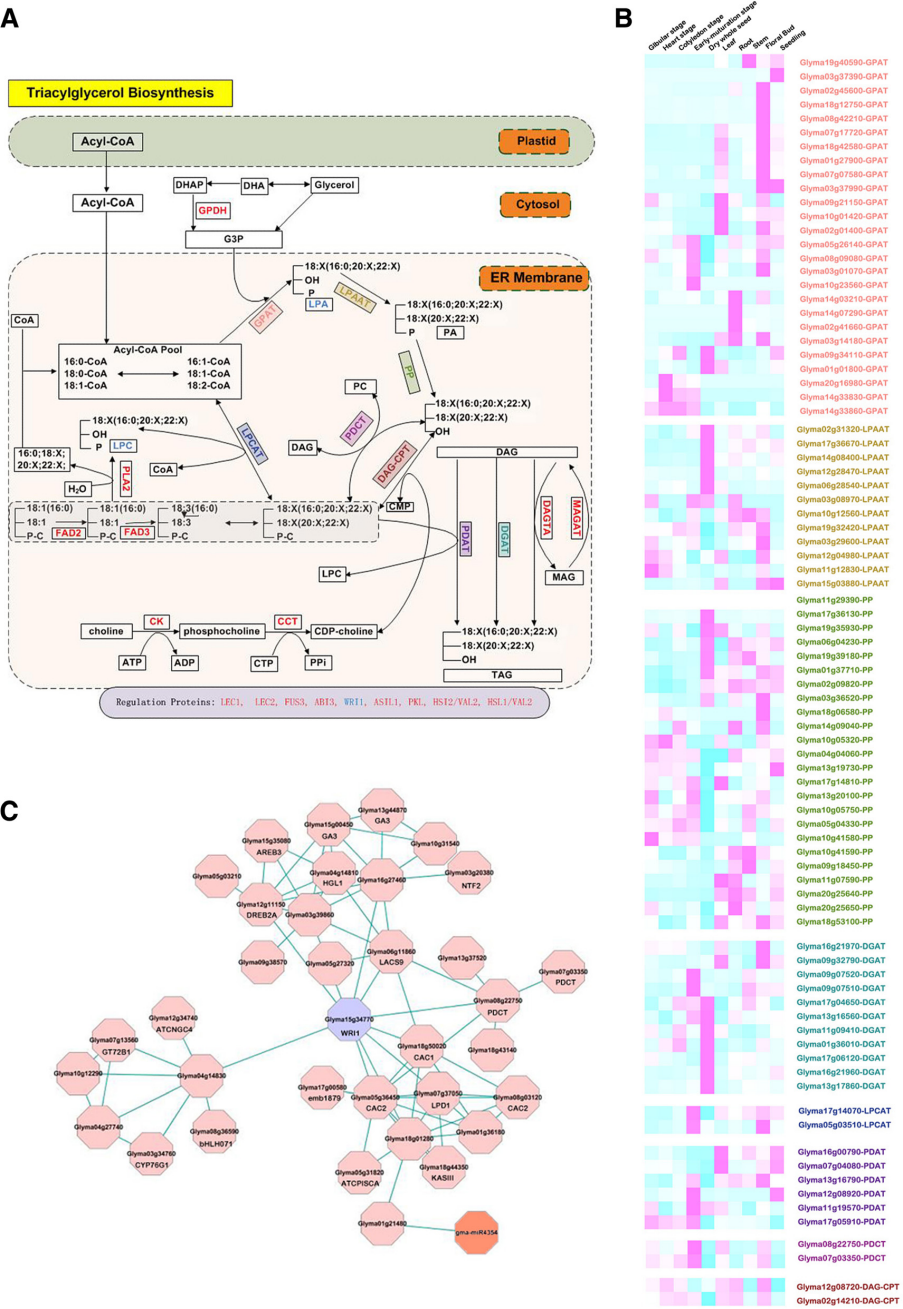
**The tissue-specific gene expression and regulation network of the triacylglycerol (TAG) biosynthesis pathway.** Multiple genes of interest in the TAG biosynthesis pathway **(A)** are simultaneously shown in a heatmap **(B)** with the same color. The co-expression network of WRI1 (Glyma15g34770) (C), which regulates the biosynthesis of TAG.

Here, we selected the biosynthesis pathway of triacylglycerol (TAG) as an example to introduce the application of our database. Figure 1A shows the biosynthesis pathway of TAG and Figure 1B shows the tissue-specific expression of genes that encode the enzymes involved in this pathway–all these data can be obtained from the SFGD platform. Additional file 4: Figure S1 shows a screenshot of the web page with brief information (a coding gene, the corresponding probe set, GmaAffx.88235.1.S1_at and one microarray expression series, and ten deep sequence samples). Figure 1C shows the co-expression network of WRI1 (Glyma15g34770), which has been reported to regulate the process of TAG biosynthesis by affecting the synthesis precursors in developing embryos [77,78]. Table 4 lists all soybean coding genes that appeared in Figure 1C and their annotations, and also gives these genes’ orthologs in *Arabidopsis*. Users can also do cis-element significance analysis, and a job ID is produced by the server–the results are stored in the server for several weeks so that they can be traced back. The resulting web-page only shows motifs with P < 0.05, and Table 5 is one example of the significance analysis results for all genes that appeared in Table 4. Table 5 shows that some motifs may be closely related to seed development, such as ‘ABREBNNAPA’ and ‘ABRETAEM’, with their annotations ‘napA; storage protein; ABRE; napin; seed’ and ‘ABA; ABRE; EMBP-1; seed’, respectively.

**Table 4:** Soybean lipid biosynthesis related genes (WRI1 network genes)

**Gene**	**Probe set(s)**	**Homolog**	**Annotation**
Glyma06g11860	GmaAffx.50807.1.S1_at, GmaAffx.50807.2.S1_at, GmaAffx.26813.1.A1_at	AT1G77590	LACS9 (LONG CHAIN ACYL-COA SYNTHETASE 9); long-chain-fatty-acid-CoA ligase
Glyma18g50020	Gma.16819.1.S1_at	AT5G16390	CAC1 (CHLOROPLASTIC ACETYLCOENZYME A CARBOXYLASE 1); acetyl-CoA carboxylase/biotin binding
Glyma07g37050	GmaAffx.84778.1.S1_at	AT3G16950	LPD1 (LIPOAMIDE DEHYDROGENASE 1); dihydrolipoyl dehydrogenase
Glyma08g22750	GmaAffx.47472.1.S1_at	AT3G15820	Phosphatidic acid phosphatase-related/PAP2-related
Glyma03g39860	Gma.959.1.S1_at	AT1G54860	Unknown protein
Glyma04g14830	GmaAffx.86095.1.S1_at	AT1G65870	Disease resistance-responsive family protein
Glyma18g01280	Gma.910.1.A1_at	AT1G24360	3-oxoacyl-(acyl-carrier protein) reductase, chloroplast/3-ketoacyl-acyl carrier protein reductase
Glyma05g36450	Gma.8414.1.S1_at, Gma.8414.1.S1_s_at	AT5G35360	CAC2; acetyl-CoA carboxylase/ biotin carboxylase
Glyma12g11150	GmaAffx.3734.1.S1_at	AT5G05410	DREB2A; DNA binding/transcription activator/ transcription factor
Glyma16g27460	GmaAffx.25251.1.S1_at	AT2G39210	nodulin family protein
Glyma01g21480	GmaAffx.85056.2.S1_at, Gma.14628.1.S1_at	AT1G25510	aspartyl protease family protein
Glyma15g35080	Gma.12634.1.A1_s_at	AT3G56850	AREB3 (ABA-RESPONSIVE ELEMENT BINDING PROTEIN 3); DNA binding/transcription activator/transcription factor
Glyma12g34740	Gma.11970.1.S1_at	AT5G54250	ATCNGC4 (CYCLIC NUCLEOTIDE-GATED CATION CHANNEL 4); calmodulin binding/cation channel/cation transmembrane transporter/cyclic nucleotide binding
Glyma15g00450	Gma.12064.1.S1_at	AT5G25900	GA3 (GA REQUIRING 3); ent-kaurene oxidase/oxygen binding
Glyma03g34760	GmaAffx.93571.1.S1_s_at, GmaAffx.93571.1.S1_at, GmaAffx.73653.1.S1_at	AT3G52970	CYP76G1; electron carrier/heme binding/iron ion binding/monooxygenase/oxygen binding
Glyma01g36180	Gma.3792.1.A1_at	AT1G42960	unknown protein
Glyma10g31540	Gma.17374.1.S1_at, GmaAffx.64124.1.S1_at, GmaAffx.87290.1.S1_at	AT1G32900	starch synthase, putative
Glyma04g27740	GmaAffx.25768.1.S1_at	AT1G65870	disease resistance-responsive family protein
Glyma08g03120	Gma.181.1.S1_at	AT5G35360	CAC2; acetyl-CoA carboxylase/ biotin carboxylase
Glyma05g03210	Gma.8701.1.S1_at, GmaAffx.7258.1.S1_s_at	AT4G24830	arginosuccinate synthase family
Glyma18g43140	Gma.2316.1.S1_at	AT5G05600	oxidoreductase, 2OG-Fe(II) oxygenase family protein
Glyma07g13560	GmaAffx.5167.1.S1_at	AT4G01070	GT72B1; UDP-glucosyltransferase/UDP-glycosyltransferase/transferase, transferring glycosyl groups
Glyma09g38570	GmaAffx.81233.1.A1_at	AT5G16460	Unknown protein
Glyma10g12290	Gma.1883.1.S1_at	AT2G41190	Amino acid transporter family protein
Glyma08g36590	Gma.14186.1.A1_at	AT5G46690	bHLH071 (beta HLH protein 71); DNA binding/transcription factor
Glyma13g44870	GmaAffx.59734.1.A1_at, GmaAffx.86023.1.S1_at, GmaAffx.33541.1.S1_at	AT5G25900	GA3 (GA REQUIRING 3); ent-kaurene oxidase/oxygen binding
Glyma03g20380	GmaAffx.37979.1.S1_at	AT3G07250	Nuclear transport factor 2 (NTF2) family protein/RNA recognition motif (RRM)-containing protein
Glyma05g31820	GmaAffx.29450.1.S1_at	AT1G10500	ATCPISCA (chloroplast-localized IscA-like protein); structural molecule
Glyma17g00580	Gma.10258.1.A1_s_at, Gma.10258.2.S1_at, GmaAffx.83788.1.S1_at	AT5G49820	emb1879 (embryo defective 1879)
Glyma07g03350	Gma.11469.1.S1_at, GmaAffx.67403.1.S1_at, GmaAffx.67403.1.A1_at	AT3G15820	phosphatidic acid phosphatase-related/PAP2-related
Glyma18g44350	Gma.6041.1.S1_at	AT1G62640	KAS III (3-KETOACYL-ACYL CARRIER PROTEIN SYNTHASE III); 3-oxoacyl-[acyl-carrier-protein] synthase/catalytic/transferase, transferring acyl groups other than amino-acyl groups

**Table 5:** Motif significance analysis results of soybean triacylglycerol biosynthesis related genes

**Motif**	**Factor**	**Count**	**ZFM**	**P-value**	**Keywords**
GTCATTATCGG	CATTAT-motif	1	14.1	0	phyA3;Avena sativa
CGCCACGTGTCC	ABREBNNAPA	2	10.81	0	napA; storage protein; ABRE; napin; seed
AATTAAA	POLASIG2	453	8.42	0	poly A signal
GGACACGTGGC	ABRETAEM	3	5.87	0	ABA; ABRE; EMBP-1; seed
ACGTGKC	ACGTABREMOTIFA2OSEM	30	5.17	0	ABA; ABRE; motif A; DRE
MCACGTGGC	GBOXLERBCS	9	5.17	0	G box; rbcS; tomato; G-box; leaf; shoot
ATTAAT	Box 4	664	4.7	0.000001	pal-CMA1;light responsiveness
TCCACGTGGC	LREBOXIIPCCHS1	3	4.69	0.000001	Chalcone synthase; CHS; light; Box II; LRE; leaf; shoot
GTATGATGG	SORLIP4AT	4	4.63	0.000002	phyA; phytochrome; light
YACGTGGC	ABREATCONSENSUS	11	4.38	0.000006	ABA; ABF; bZIP factors
ACGTGGC	BOXIIPCCHS	15	4.35	0.000007	Box II; Box 2; CHS; chs; light regulation
CACGTGGC	EMBP1TAEM	9	4.24	0.000011	EMBP-1; Em; ABA; ABF; ABRE; bZIP; seed
TGTATATAT	SORLREP3AT	43	4.13	0.000018	phyA; phytochrome; light
CCNNNNNNNNNNNNCCACG	UPRMOTIFIIAT	8	3.96	0.000037	UPR; unfolded protein response
TCCACGTGTC	SGBFGMGMAUX28	2	3.85	0.000058	Aux28; G box; auxin; bZIP; SGBF-1; SGBF-2
AGATATGATAAAA	IBOXLSCMCUCUMISIN	1	3.79	0.000075	Cucumisin; fruit
ACGTGGCA	LRENPCABE	8	3.68	0.000117	CAB; cab; cab-E; CABE; light; leaf; shoot
CACGTGG	IRO2OS	16	3.55	0.00019	root; shoot; Fe; iron
CCACGTGG	ABREZMRAB28	8	3.54	0.000201	Freezing tolerance; seed; shoot; CBF2
ACGTGTC	GADOWNAT	15	3.35	0.000404	Ga; seed; germination
GCCACGTGGC	ACGTROOT1	2	3.12	0.000898	Root; ACGT; G box; G-box; ABRE motif; bZIP binding enhancement
ACGTCA	HEXMOTIFTAH3H4	33	2.83	0.002325	Leucine zipper motif; meristem; OBF1; bZIP; lip19; LIP1
TGACGT	TGACGTVMAMY	33	2.83	0.002325	Alpha-Amylase; cotyledon; seed germination; seed
CACGTG	CACGTGMOTIF	44	2.83	0.002359	G box; G-box; rbcs; chs; ACGT element; adh; Bz-2; R-motif; STR;GT-1; GBF; elicitor; bZIP; napin; strictosidine synthase; cell
TGTAATAATATATTTATATT	Unnamed__5	5	2.77	0.002822	SEF1 factor binding site;seeds
AATTATTTTTTATT	AT1-motif	4	2.5	0.006162	Light responsive element
CCACGTGGCC	CPRFPCCHS	1	2.48	0.006584	BoxII; CPRF; bZIP; leaf; shoot; CHS; ACE; light; bZIP
CCWWWWWWWWGG	CARGNCAT	16	2.45	0.007059	MADS; AGAMOUS; AGL; embryo
RYACGTGGYR	ABREATRD22	5	2.34	0.009586	ABA; responsive element; ABRE; rd22; RD22; dehydration; shoot

Note: ZFM (Z-score for motif) and P-value are described in ‘Cis-element significant analysis’ section(job ID:job2014Mar4201558, produced by inputting genes appearing in Table 4).

### More function modules

Home page: this describes the whole database project and other soybean resources.

General search page: SFGD supports fuzzy queries by one or a list of gene names, flcDNA IDs, probe set IDs or function keywords. Users can convert gene names to corresponding probe set names by input of a list of gene names, and vice-versa.

BLAST search: a BLAST search toolbox is provided and users can do BLAST searches against soybean cDNA, flcDNA, probe set consensus and protein sequence.

Download: users can download all coding genes’ sequences and their annotations. Full-length cDNA sequences, consensus sequences and annotations appear in the database.

**Figure 2:**
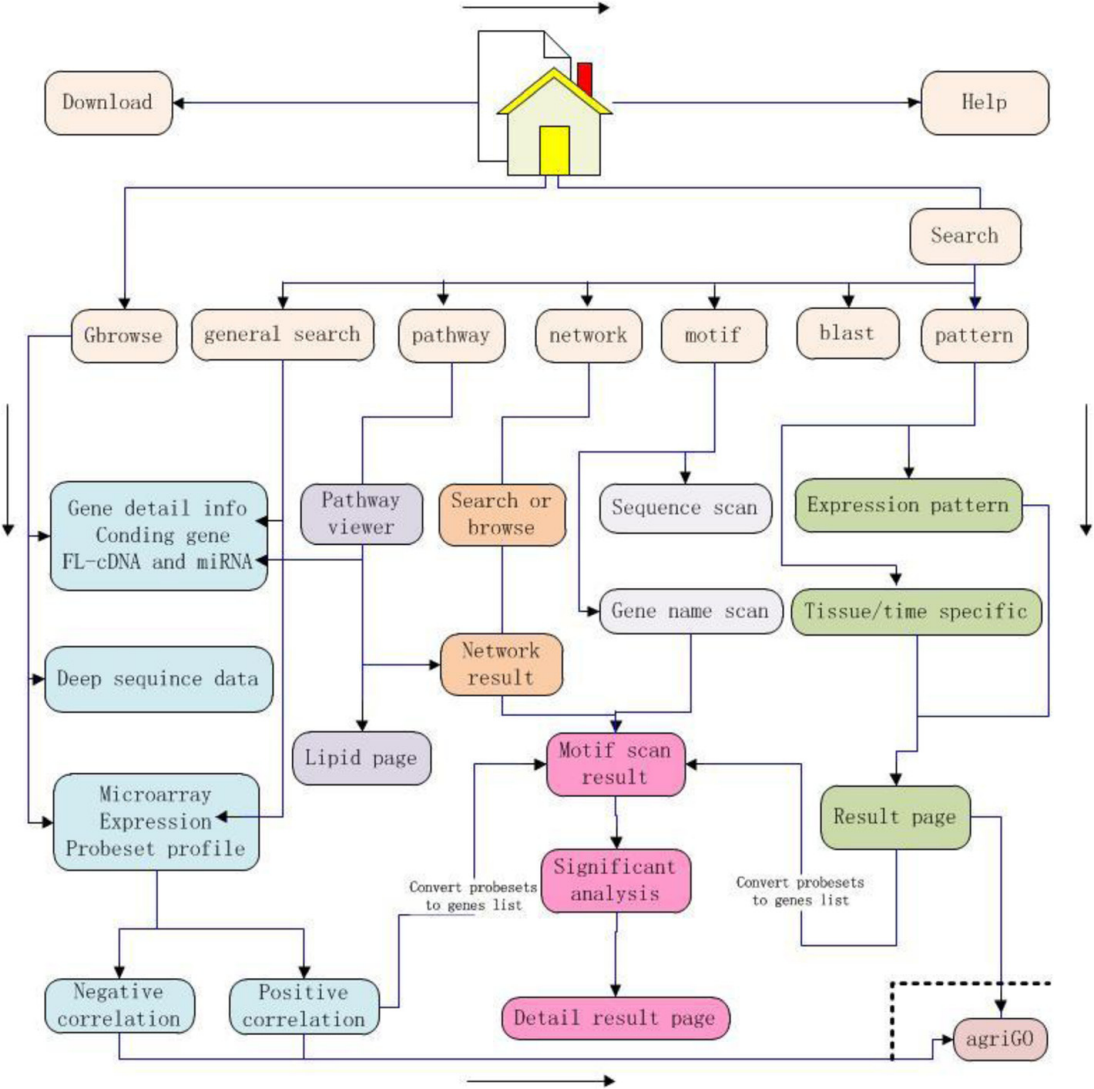
**Structure of the SFGD database.** Rectangles with rounded corners are pages in the database, and the directed lines show linkage for pages.

The SFGD was constructed using Hypertext Markup Language (HTML), perl CGI (http://www.perl.com) and the MySQL 4.0 (http://www.mysql.com) database engine. Figure 2 shows an overview of our websites. The whole SFGD database is run on a server using the LINUX operating system.

## Discussion

There is no doubt that a novel and comprehensive method of integration and analysis for soybean omics data will provide valuable insights and help to improve soybean. However, a powerful mining tool for omics data is limited. To fill the gap of a platform for acyl-lipid comprehensive analysis and information for high-throughput omics data, we developed SFGD–a comprehensive and integrated database for mining functional information from soybean transcriptome data and identifying acyl-lipid metabolism pathways. This database include a variety of information, for instance, microarray expression data with 255 samples from 14 experiments, 30 mRNA-seq samples belonging to four experiments, other genomic data including coding and non-coding genes (miRNA), as well as a set of SNPs from 17 wild and 14 cultivated soybean sub-species [52]. By integration and analysis of these data, we provide a one-stop-shop resource for acyl-lipid researchers, we also integrated the genome and transcriptome information into Gbrowse, which can provide the tissue and time points for specific expression information. We then built some function toolboxes: e.g. gene co-expression network, cis-element significance analysis tool, expression pattern search module and some other functions.

As an example of TAG biosynthesis in the previous description (Figure 1A), Gbrowse provides gene expression information on various tissue types and stress conditions. From these data, we can infer the main place, time or possible function for a specific enzyme (Figure 1B).

The biosynthesis of TAG occurs at the endoplasmic reticulum and probably also involves reactions at the oil body [79]. The classical pathway of TAG biosynthesis in seeds is the acyl-CoA dependent Kennedy pathway (or the glycerol phosphate pathway)–this pathway consists of sequential acylation and subsequent dephosphorylation of glycerol-3-phosphate (G3P). The first acylation of G3P is catalyzed by glycerol-3-phosphate acyltransferase (GPAT; EC 2.3.1.15). GPAT was first cloned from *Saccharomyces cerevisiae*; and a member of the *Arabidopsis* GPAT gene family displayed GPAT activity when expressed in yeast. However, mutations of GPAT gene in *Arabidopsis* do not appear to affect seed oil level, but play a role in production of cutin and suberin [80-82]. In our search through the transcriptome profiling data, we found few GPAT family genes were highly expressed in seed at the development stage. Of course, this does not suggest that there is no relationship between GPAT family and seed oil level. Genes encoding GPAT9 are most likely to be important in soybean oil synthesis and have significantly higher expression in seeds at different stages. Similar results were also reported in castor [83].

The second acylation is catalyzed by 1-acylglycerol-3-phosphate acyltransferase (LPAAT; EC 2.3.1.51). A number of LPAAT genes are highly expressed in the dry whole seed, suggesting involvement in the storage rather than the synthesis stage of oil metabolism.

Phospatidate phosphatase (PP; EC 3.1.3.4) catalyzes the dephosphorylation of phosphatidic acid to form *sn*-1,2-diacylglycerol [84]. A variety of PP types and isoforms exist in plants but their exact role in TAG biosynthesis is unclear. In our analysis results, we found that some PP genes were highly expressed in dry seeds–e.g. PHA1 (Glyma19g35930), PHA2 (Glyma 17 g36130, Glyma06g04230) and LCBCP (Glyma19g39180)–these genes will be candidates for further study.

The final acylation reaction, converting diacylglycerol (DAG) to TAG is catalyzed by enzymes. In *Arabidopsis*, DGAT1 has been shown to play a role in seed oil accumulation [85-87], and has also been reported as a key enzyme determining oil content and composition in maize [88]. In our database, most DGAT genes were highly expressed in seeds and especially dry whole seeds. Therefore, we predict that DGAT is a crucial enzyme for oil production of soybean–a suggestion also supported by previous study [7].

In acyl-CoA independent TAG synthesis pathway, lysophosphatidylcholine acyltransferase (LPCAT; EC 2.3.1.23) activity controls the regeneration of phosphocholine (PC) from lyso-PC. In our dataset, two members are highly expressed in floral buds and early-maturation seed, suggesting that the enzyme plays a role in early stages of oil synthesis but not during the storage phase.

DAG can also be acylated using PC as the acyl donor by a phospholipid:diacylglycerol acyltransferase (PDAT; EC 2.3.1.43). PDAT activity has been discovered in yeast and plants’ developing oil seeds [84]. PDAT1 and DGAT1 have overlapping functions in TAG biosynthesis in developing seeds, and the absence of DGAT1 is evidently compensated by PDAT1. However, the degree that each enzyme contributes to TAG biosynthesis in developing seeds is unknown. In our dataset, some PDAT genes are highly expressed in early maturation-stage seed, and others are highly expressed in seedlings and leaves, suggesting that in other tissues, PDAT-acylated TAG synthesis plays an important role.

DAG can also be converted to phosphatidylcholine (PtdC) via the action of phosphatidylcholine:diacylglycerol cholinephosphotransferase (PDCT) or *sn*-1,2-diacylglycerol:cholinephosphotransferase (CPT). It has been reported in soybean seeds that about 60% of newly synthesized acyl chains directly incorporate into the *sn*-2 position of PC through an acyl-editing mechanism rather than a pathway for sequential acylation of G3P [89]. PDCT has a clear seed-specific expression, but DAG-CPT does not have a clear tissue-expression–this does not immediately suggest a major role for DAG-CPT in tri-ricinolein synthesis as reported in castor [83].

In our gene co-expression regulation network, we use PCC and MR [18] values to mark the relationship between each gene (probe set). Many transcription factors are involved in a complex network to regulate TAG production [90,91]. The mutants for WRINKLED1 produce wrinkled seeds with severe depletion of TAGs [77,78], which can also be triggered by other transcription factors, such as LEC2, FUS3 and ABI3 [92,93]–with our co-expression network tool, their co-expression interaction can also be found. In addition, some other transcription factors were detected in this co-expression network (Table 4), such as AREB3, NF-YB6 and MYB65, suggesting that they may participate in the regulated network for TAG synthesis. We also compared our results with the soybean gene network in PlaNet [27] (Additional file 6: Table S4). The Glyma15g34770 network in PlaNet lists 37 genes co-expressed with WRI1, and there are 33 genes in our network, and seven of them overlap with results from PlaNet, and form the ‘band 7 family’, ‘LACS9’, ‘DREB2A’, ‘AREB3’, ‘CYP76G1’ and so on. There are some differences between these two web sites, mainly due to the diversity of microarray expression experiments used in these two web services: e.g. PlaNet’s microarray expression experiments are all tissue-specific treatments (Additional file 7: Table S5, these treatments from the top three microarray expression experiments appear in Additional file 3: Table S1), and we included some other experiments, such as time series experiments, gene mutant experiments and plant responses to biotic and abiotic stresses.

Table 5 shows the significance analysis results of motifs for all genes that appeared in Table 4, and some keywords have a close relationship with seed development, such as motif ‘ABREBNNAPA’, which has been reported as conserved in many storage-protein gene promoters of seeds [94-97] and motif ‘ABRETAEM’ play a role in seed gene expression and response to ABA [98,99]. This information gives some indication of interaction between motif and function.

The advantages of microarray expression experiments are the maturation of this technology, high-throughput and many types of analysis software. There are some disadvantages of microarray experiments in that they do not cover all soybean genes, microarrays cannot identify new genes and there may be errors when expression levels are close to background signals. Thus the next deep sequencing experiments may play important roles in constructing the gene network.

## Conclusions

Currently we have integrated soybean genome data, full-length cDNA, microarray expression experiments, deep sequencing data, miRNA precursor and SNP information into our database (Table 2). We have also developed a soybean gene co-expression regulatory network web service, a graphical display of acyl-lipid metabolic pathways, a cis-element significance analysis toolbox, an expression pattern for positive/negative search function modules and other general tools in the database. We hope this will improve the accuracy and robustness of soybean functional genomics analysis, and further hasten understanding of the gene regulatory networks for effective crop improvement. SFGD is freely available at http://bioinformatics.cau.edu.cn/SFGD/, and it will be updated every 3-6 months with the development of soybean research; however, the update must be performed by the administrator.

## Availability and requirements

The database is available at http://bioinformatics.cau.edu.cn/SFGD/ and is compatible with most modern web browsers. The user’s browser must have JavaScript enabled to show query examples and Cookie and Flash to show the expression curves.

## Competing interests

The authors declare that they have no competing interest.

## Authors’ contributions

ZZ performed data collection and annotation, and the database and web server construction; JY collected and processed RNA-seq data; and JY and JW constructed the soybean acyl-lipid metabolic pathways. ZZ and JY compiled the main parts of the manuscript. YL provided the system support. ZS and WX supervised the project. All authors read and approved the final manuscript.

## Supplementary Material

Additional file 1: Table S3 Motifs in all soybean gene promoter sequences. Click here for file

Additional file 2: Figure S2 The probe sets with positive or negative correlation with ‘GmaAffx.88235.1.S1_at’ in the experiment ‘GSE7511’. This figure includes four sub-graphs, and each shows 60 probe sets most positively or negatively correlated with probe set ‘GmaAffx.88235.1.S1_at’ within the treatment ‘GSE7511 (Expression data from soybean seed compartments with embryos at the heart stage)’. The left two sub-graphs were generated using expression values, and the right two sub-graphs were produced using ZFE (Z-score for expression), the upper two sub-graphs show probe sets positively correlated with ‘GmaAffx.88235.1.S1_at’, and the lower two sub-graphs shows probe sets negatively correlated with ‘GmaAffx.88235.1.S1_at’. Click here for file

Additional file 3: Table S1 Microarray expression experiments and deep-seq experiments collected by SFGD. Click here for file

Additional file 4: Figure S1 Snapshot of Gbrowse in SFGD database for WRI1 (Glyma15g34770). This is a snapshot of Gbrowse (genome viewer) in our database, here we use WRI1 (Glyma15g34770), a lipid synthesis related gene as a sample, and it also shows one microarray expression experiment and 10 deep sequences as evidence of this gene. Click here for file

Additional file 5: Table S2 Top 10 genes with most edges in our comprehensive soybean gene network. Click here for file

Additional file 6: Table S4 Network comparison between SFGD and PlaNet. Click here for file

Additional file 7: Table S5 Experiments selected in PlaNet. Click here for file
