# Supplementary material for: SFGD: a comprehensive platform for mining functional information from soybean transcriptome data and its use in identifying acyl-lipid metabolism pathways
Source: BMC Genomics. 2014 Apr 8;15:271. doi: 10.1186/1471-2164-15-271 (PMC4051163; doi:10.1186/1471-2164-15-271)
Supplement: Additional file 2: Figure S2 — The probe sets with positive or negative correlation with ‘GmaAffx.88235.1.S1_at’ in the experiment ‘GSE7511’. This figure includes four sub-graphs, and each shows 60 probe sets most positively or negatively correlated with probe set ‘GmaAffx.88235.1.S1_at’ within the treatment ‘GSE7511 (Expression data from soybean seed compartments with embryos at the heart stage)’. The left two sub-graphs were generated using expression values, and the right two sub-graphs were produced using ZFE (Z-score for expression), the upper two sub-graphs show probe sets positively correlated with ‘GmaAffx.88235.1.S1_at’, and the lower two sub-graphs shows probe sets negatively correlated with ‘GmaAffx.88235.1.S1_at’. [file 1471-2164-15-271-S2.DOC]

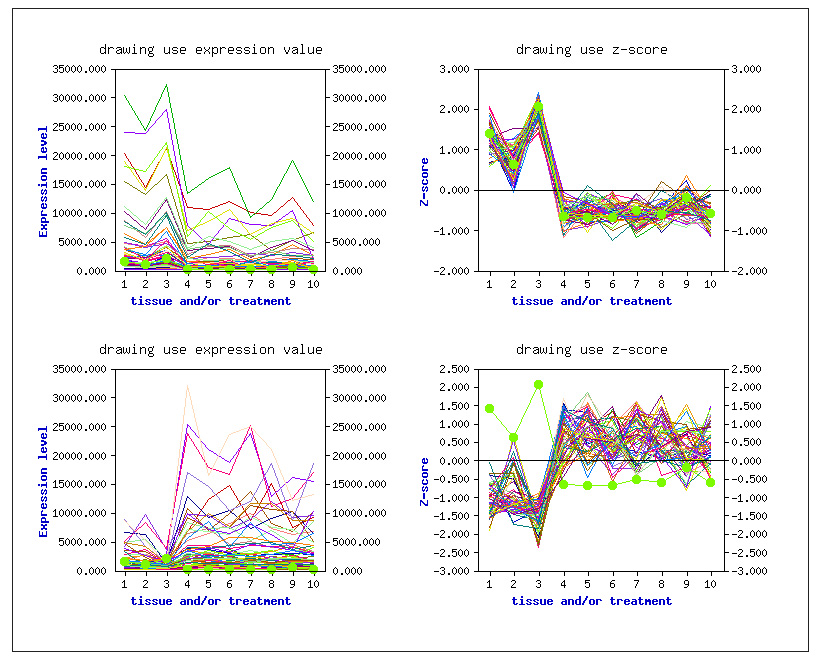


**Additional File 2: Figure S2:** This figure includes four sub-graphs, each shows 60 probe sets most positively or negatively correlated with probe set ‘GmaAffx.88235.1.S1_at’ within the treatment ‘GSE7511 (Expression data from soybean seed compartments with embryos at the heart stage)’. The left two sub-graphs were generated using expression values, and the right two sub-graphs were produced using ZFE (Z-score for expression), the upper two sub-graphs show probe sets positively correlated with ‘GmaAffx.88235.1.S1_at’, and the lower two sub-graphs shows probe sets negatively correlated with ‘GmaAffx.88235.1.S1_at’.
