# Supplementary material for: SFGD: a comprehensive platform for mining functional information from soybean transcriptome data and its use in identifying acyl-lipid metabolism pathways
Source: BMC Genomics. 2014 Apr 8;15:271. doi: 10.1186/1471-2164-15-271 (PMC4051163; doi:10.1186/1471-2164-15-271)
Supplement: Additional file 4: Figure S1 — Snapshot of Gbrowse in SFGD database for WRI1 (Glyma15g34770). This is a snapshot of Gbrowse (genome viewer) in our database, here we use WRI1 (Glyma15g34770), a lipid synthesis related gene as a sample, and it also shows one microarray expression experiment and 10 deep sequences as evidence of this gene. [file 1471-2164-15-271-S4.DOC]

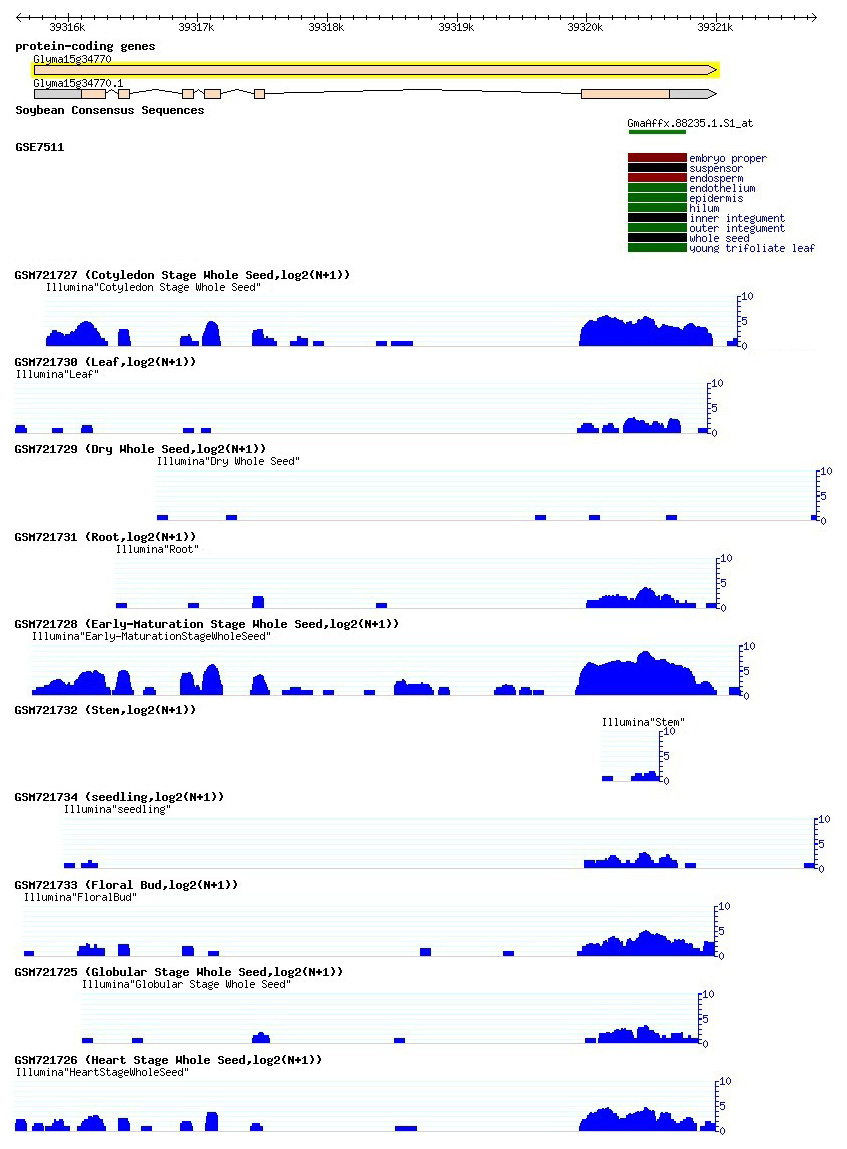


**Additional File 4: Figure S1:** This is a snapshot of Gbrowse (genome viewer) in our database, here we use WRI1 (Glyma15g34770), a lipid synthesis related gene as a sample, and it also shows one microarray expression experiment and 10 deep sequence evidence of this gene.
